# Cross-cultural validation and psychometric testing of the Debriefing Experience Scale (DES): a cross-sectional study

**DOI:** 10.1186/s12909-022-03332-8

**Published:** 2022-04-13

**Authors:** Ya Dian Xie, Xin Yi Li, Qian Liu, Run Huang, Ting Li, Ya Xuan Fang, Dan Luo, Yonghui Wan, Bing Xiang Yang, Shelly J. Reed

**Affiliations:** 1 School of Nursing, Wuhan University, No.115 Donghu Road, Wuhan, 430071 China; 2 Teaching Office, Zhongnan Hospital of Wuhan University, Wuhan, China; 3 Sir Run Run Shaw Hospital, School of Medicine, Zhejiang University, Hangzhou, Zhejiang 310016 China; 4 School of Nursing, Southern Medical University, Guangzhou, China; 5 Oncology Center, Renmin Hospital of Wuhan University, No.238 Jiefang Road, Wuhan, 430060 China; 6 College of Nursing, Brigham Young University, Provo, UT USA

**Keywords:** Debriefing, Simulation-based education, Nursing education, Debriefing experience scale

## Abstract

**Background:**

The Debriefing Experience Scale (DES) is a tool that is used to explore nursing students’ subjective experiences during a debriefing and to help determine best debriefing practices. A Chinese version of the scale has not been found; its development can enhance learning in simulation activites in Chinese healthcare education programs.

**Methods:**

A simplified Chinese version of the DES was developed and tested using 34 Chinese undergraduate (second year) nursing students. They participated in six simulation scenarios and debriefings. Eight experts were consulted to determine the content validity of the scale. Critical ratio method, Cronbach’s alpha, intraclass correlation coefficient, correlation coefficient and factor analysis were used in testing the psychometric properties of the scale.

**Results:**

Analysis of 200 scales showed that the simplified Chinese version of the DES had good potential in discriminatiing Chinese nursing students’ experiences of debriefing.

**Conclusions:**

The simplified Chinese DES was effective in evaluating the experience of debriefing. A larger sample size and multicenter research is needed to confirm these findings.

**Supplementary Information:**

The online version contains supplementary material available at 10.1186/s12909-022-03332-8.

## Introduction

Simulation-based education is a teaching strategy that can improve clinical competency of health care professionals [1, 2]. Jeffries [3] described the three phases of simulation as pre-briefing, scenario and debriefing. The final phase of debriefing is the act of reviewing critical actions that unfolded during the course of a simulation scenario [4]. During debriefing, faculty and students can reflect on the simulation experience from a variety of perspectives, exchange feedback and review performance errors [5, 6]. Debriefing is considered as a vital factor in simulation-based education and can provide opportunities to improve clinical performance [7–10], because the quality of a debriefing determines the effectiveness of simulation education [11]. In order to determine the quality and best practices for debriefing, various instruments have been developed [12–16].

As debriefing is conversational, bidirectional, interactive and reflective [17], the experience and evaluation of students in debriefing is important. There are some instuments for debriefing evaluation. For example, the Objective Structured Assessment of Debriefing is a often-reported tool to assess a debriefer’s performance [12]; the Debriefing Assessment for Simulation in Healthcare evaluates debriefing by examining debrefier’s concrete behaviors [13]; while the Peer Assessment Debriefing Instrument could be used as a self and peer assessment in evaluation [14]. But there is still a lack of knowledge about the perceptions of students regarding the debriefing experience [15]. Reed [15] developed the Debriefing Experience Scale (DES) to explore nursing students’ subjective experiences during debriefing. In Reed’s research, a validation study of this scale was carried out with 130 nursing students. The results showed that the internal consistency reliability, measured by Cronbach’s alpha, was reported to be 0.93 for the experience scale and 0.91 for the importance scale [15]. The scale has been translated into Norwegian [18] and Portuguese [19], and these translations have shown good psychometric properties and potential for use. There is no report of a Chinese version of the DES that has been verified for reliability and validity.

This study aimed to translate the DES into a Simplified Chinese version, and determine its reliability and validity.

## Methods

### Study design

The study is an instrument adaption with psychometric testing. A cross-sectional study design was used.

### Instruments: debriefing experience scale

The Debriefing Experience Scale (DES) was developed by Reed [15] to measure (a) the experience of students during debriefing and (b) the importance of these experiences to the student. The DES has 20 items which are divided into four subscales. For each item, study participants were asked to evaluate both the student experience and the perceived importance of the item using a five-point Likert-type rating scale. The experience scale was rated from 1 (strongly disagree) to 5 (strongly agree), including the alternative of not applicable (NA), that is, the statement had nothing to do with the reporting activities carried out. The importance scale was rated from 1 (not important) to 5 (very important).

### Setting

This study was conducted at the School of Nursing, Wuhan University, China, which is considered as a demonstration and training center for simulation-based education in nursing. The facilitators were certified as a Simleader by the National League for Nursing (NLN), and have completed the standardized training courses developed by the NLN. These courses include Foundations in Simulation, Debriefing Foundations, Curriculum Integration and Evaluation. Faculty of the nursing program who are facilitators have adopted the International Nursing Association for Clinical Simulation and Learning [20] Standards of Best Practice [20] in implementing simulation-based education. Their debriefings follow the Gather-Analysis-Summary (GAS) model [21], which is a learner-centered process.

The simulation experiences of nursing students in this study were part of a compulsory baccalaureate nursing course that focused on integration of knowledge in developing clinical skills. Students participated in three simulations related to psychiatric nursing: a client having auditory hallucinations, managing a client who is violent and impulsive, and suicide crisis intervention. These simulations were conducted by SimLeader A. The other three simulations related to medical-surgical nursing and focused on a client having a cast, care of a client in traction and care of a client with complications of a fracture. These were led by SimLeader B. Students were divided into four groups (eight to nine students per group) and each group would participate in the six simulations.

### Sample

A convenience sample was used for the study. None of the researchers participated in simulation activities in the nursing education program. After an introduction to the research study and its purpose, 34 s year baccalaureate nursing students agreed to participate. Five were male (14.7%) and 29 were female (85.3%), ranging in age from 19 to 21 years, with an average age of 19.94 $\pm$ 0.42 years; and, they were from three provinces in China.

### Procedures and data collection

**Fig. 1:**
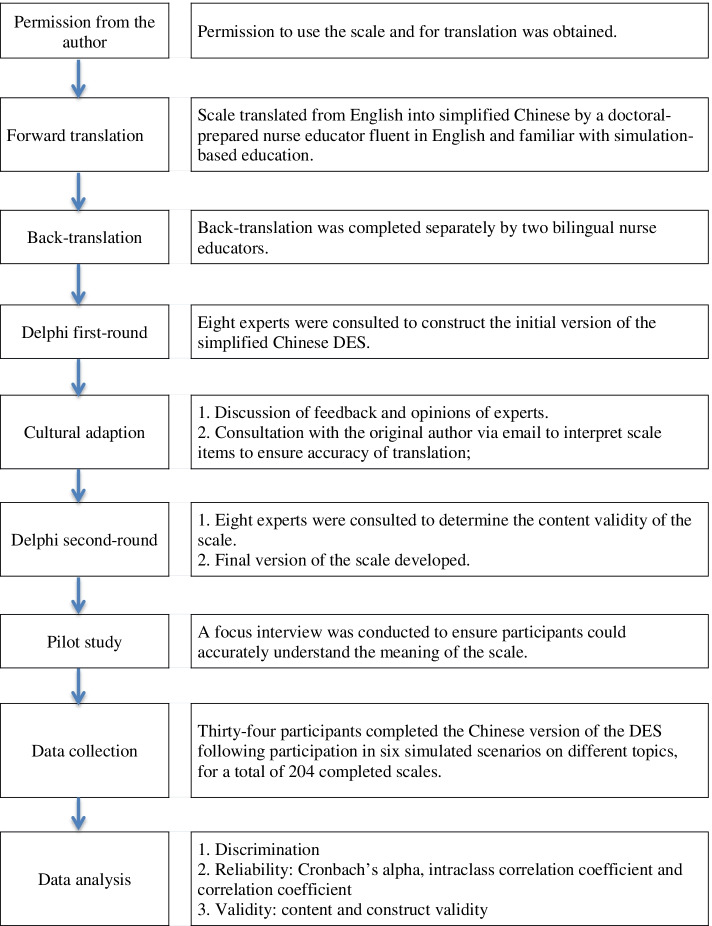
Study procedures

After permission was obtained from the original author, the DES was translated into simplified Chinese based on standardized guidelines [22] including forward translation, back translation, cultural adaption and pilot testing. A focus interview with one group of participants was conducted to ensure that participants could understand the meaning of the scale. Data were collected using the translated Simplified Chinese version of the DES. Participants rotated through the six simulations during one semester (4 months) of their nursing program and completed the scale following each debriefing experience. A total of 204 scales were completed for a return rate of 100%. After excluding four invalid scales due to data missing, 200 were included in the analysis. Testing of items included discrimination and the reliability and validity of the scale. Study procedures are shown in Fig. 1.

### Ethics

This study was approved by the Medical Ethics Committee of Wuhan University School of Medicine in Wuhan, China (NO. 2021YF0002). Participants completed informed consent and were allowed to withdraw at any time. Researchers iterated via oral and written means that confidentiality was maintained, a student’s participation would not affect their grade in the course in which the simulations were conducted and their responses to the scales would not be shared with faculty leading the simulations.

### Data analysis

Data were analyzed using IBM SPSS Statistics, version 24 (IBM Corporation, Armonk, NY) and Amos Graphics, version 22 (IBM Corporation, Armonk, NY). The critical ratio method was used for discrimination. The scale’s total score was sorted according to the level, and the scales were divided into a high score group (top 27%), a low score group (bottom 27%) and other groups. The difference in the average score of items between the high score and the low score groups was obtained by an independent sample t-test (two-tailed probability) to judge whether the item had good discrimination. Cronbach’s alpha and the correlation coefficient between each item and the total score of the scale were used to establish the reliability of the experience and the importance scale. Intraclass correlation coefficient (ICC) was used to reflect the test–retest reliability of repeated data collection from participants; estimates and their 95% confident intervals (CI) were calculated based on a 2-way mixed-effects model [23]. Content validity was established by a group of eight experts, including four simulation and medical education experts and four simulation and nursing education experts. Exploratory factor analysis (EFA) and confirmatory factor analysis (CFA) were used to evaluate construct validity.

## Results

### Translation and culture adaption

Experts were consulted to develop the simplified Chinese version of the DES, and valuable suggestions and opinions were offered by Dr. Reed, author of the scale. The original scale format was maintained, with some changes such as adding adjectives and qualifiers to improve understanding. As a result, the word “meaning” in item four was changed to “more understanding of the topic”; the word “question” in item five was described as “questions arose in the simulation”; the word “problems” in item seven was described as “clinical problems”; the word “unsettled” in item 12 was deleted as a result of the focus interview. All changes were validated by the eight experts.

### Discrimination

The average score of each item of the experience scale (simplified Chinese version) ranged from 4.16 to 4.64, and the total score ranged from 74 to 100, for an average of 90.61 $\pm$ 6.36. The average score of the high score group (top 27%, *n* = 62) was 97.40 $\pm$ 1.83, and that of the low score group (bottom 27%, *n* = 60) was 82.59 $\pm$ 3.70. The results of independent sample t-test showed that the difference between the two groups was statistically significant at the 0.05 level (*t* = 27.89, *p* < 0.01).

The average score of each item in the importance scale (simplified Chinese version) was 4.08 to 4.62 and the total score was from 71 to 100, with an average score of 88.62 ± 7.25. The average score of the high score group (top 27%, *n* = 60) was 97.02 ± 2.15, and it was 79.27 ± 2.84 in the low score group (bottom 27%, *n* = 55). The results of independent sample t-test showed that the difference between the two groups was statistically significant at the 0.05 level (*t* = 37.92, *p* < 0.01).

### Reliability

Cronbach’s alpha for the simplified Chinese DES measured during the six simulation cases were 0.90 (case one, *n* = 33), 0.91 (case two, *n* = 34), 0.91 (case three, *n* = 34), 0.89 (case four, *n* = 32), 0.81 (case five, *n* = 33) and 0.90 (case six, *n* = 34), respectively (see Supplemental Table 1). The test–retest reliability (ICC) was 0.86 (95% CI 0.64–0.98). When it came to the 200 scales, Cronbach’s alpha was determined for both the experience scale and the importance scale, with the Cronbach’s alpha for all items in the experience scale as 0.90, and the Cronbach's alpha for all items in the importance scale as 0.92. The subscale, Analyzing Thoughts and Feelings, exhibited an alpha value below that of the acceptable value 0.70 [24]. Alpha values for the experience and importance scale within the simplified Chinese DES, and for the subscales, are shown in Table 1.

**Table 1:** Cronbach’s alpha for the simplified Chinese DES

	Cronbach’s alpha experience items	Cronbach’s alpha importance items	Number of items in scale/subscale
Subscale			
Learning and Making Connections	0.78	0.81	8
Analyzing Thoughts and Feelings	0.65	0.66	4
Facilitator Skill in Conducting the Debriefing	0.72	0.74	5
Appropriate Facilitator Guidance	0.71	0.75	3
Overall scale	0.90	0.92	20

The correlation coefficient between each item and the total score of the experience scale was 0.48 (item 12) to 0.69 (item 9 & item 18), and 0.50 (item 12) to 0.72 (item 18) for the importance scale. The correlation coefficient between the subscales and the total score of the experience scale was 0.82 to 0.92, with that for the importance scale was 0.83 to 0.93. The correlation was significant at the 0.01 level (2-tailed) (see Table 2). The correlation coefficient between any two subscales was greater than 0.59 (0.60 to 0.79) and the correlation was significant at the 0.01 level (2-tailed).

**Table 2:** Pearson correlation coefficient between each subscale and the total score of the scale

Subscale	Total score of the experience scale	Total score of the importance scale
Learning and Making Connections	0.92	0.93
Analyzing Thoughts and Feelings	0.82	0.83
Facilitator Skill in Conducting the Debriefing	0.86	0.92
Appropriate Facilitator Guidance	0.83	0.85

Correlation is significant at the 0.01 level (2-tailed)

### Content validity

Content validity was established by a group of eight experts, four males and four females, aged between 32 and 47 years, with an average age of 37.75 $\pm$ 5.06 years; five experts had doctoral degrees. They were simulation experts in medical or nursing education and had been working for 3 to 27 (13.88 $\pm$ 7.30) years. Each item of the simplified Chinese version of the DES was rated using a Likert-type scale, from 1 (not important) to 5 (very important). The content validity index (CVI) of each item was from 0.83 to 1.00; the CVI of the total scale was 0.94.

### Construct validity

Before conducting the factor analysis, the suitability of the data for the exploratory factor analysis (EFA) was assessed.

Sampling adequacy was determined by the Kaiser–Meyer–Olkin (KMO) test of the experience scale and was found to be 0.91, and the Bartlett's Sphericity Test chi-square was 1352.87. The degree of freedom was 190, *p* < 0.01, and the anti-image matrix ranged between 0.85 and 0.94.

An initial analysis was run, and three components showed an eigenvalue above Kaiser’s criterion of 1, explaining 47.69% of the variance in the data from the experience scale (35.82%, 6.30% and 5.57% respectively). The scree plot showed a clear break after the second component.

An extraction followed by an Oblimin rotation with Kaiser normalization was conducted. The pattern matrix (see Table 3) showed that component 1 included 12 items, component 2 included five items, component 3 included two items, and item 12 “Unsettled feelings from the simulation were resolved by debriefing” was removed by showing a loading value below the acceptable value 0.40 [25]. However, in the structure matrix, several cross-loadings were shown (see Table 4). The result of this analysis was very different from the findings in the original version. No relationship among the groups was established in the EFA, thus it was decided to follow the division established by the original version of the scale.

**Table 3:** Simplified Chinese DES: pattern matrix ^a^

Scale (one item removed)	Component		
	1	2	3
Item 5: My questions from the simulation were answered by debriefing	**0.74**	-0.15	-0.07
Item 2: Debriefing was helpful in processing the simulation experience	**0.72**	0.14	0.34
Item 19: The facilitator provided constructive evaluation of the simulation during debriefing	**0.71**	-0.02	0.03
Item 10: The facilitator reinforced aspects of the health care team’s behavior	**0.68**	-0.01	0.08
Item 20: The facilitator provided adequate guidance during the debriefing	**0.59**	0.21	0.05
Item 15: Debriefing provided a means for me to reflect on my actions during the simulation	**0.56**	0.07	-0.16
Item 9: Debriefing helped me to analyze my thoughts	**0.56**	0.07	-0.32
Item 8: Debriefing helped me to make connections between theory and real-life situations	**0.54**	-0.01	-0.16
Item 11: The debriefing environment was physically comfortable	**0.52**	0.27	0.28
Item 4: Debriefing helped me to find meaning in the simulation	**0.49**	0.13	-0.16
Item 1: Debriefing helped me to make connections in my learning	**0.44**	0.13	-0.40
Item 3: Debriefing provided me with a learning opportunity	**0.42**	0.12	-0.23
Item 17: The debriefing session facilitator was an expert in the content area	-0.10	**0.74**	-0.07
Item 18: The facilitator taught the right amount during the debriefing session	0.19	**0.68**	0.06
Item 14: The debriefing session facilitator talked the right amount during debriefing	-0.02	**0.68**	-0.14
Item 6: I became more aware of myself during the debriefing session	0.03	**0.65**	0.14
Item 16: I had enough time to debrief thoroughly	0.13	**0.59**	-0.04
Item 7: Debriefing helped me to clarify problems	0.24	0.05	**-0.61**
Item 13: The facilitator allowed me enough time to verbalize my feelings before commenting	-0.01	0.46	**-0.59**

Extraction method: Principal Component Analysis

Rotation method: Oblimin with Kaiser Normalization

^a^ Rotation converged in 10 iterations

**Table 4:** Simplified Chinese DES: structure matrix

Scale (one item removed)	Component		
	1	2	3
Item 2: Debriefing was helpful in processing the simulation experience	**0.72**	**0.46**	0.14
Item 19: The facilitator provided constructive evaluation of the simulation during debriefing	0.70	0.36	-0.14
Item 20: The facilitator provided adequate guidance during the debriefing	**0.70**	**0.52**	-0.13
Item 5: My questions from the simulation were answered by debriefing	0.67	0.26	-0.21
Item 9: Debriefing helped me to analyze my thoughts	**0.67**	0.44	**-0.47**
Item 10: The facilitator reinforced aspects of the health care team’s behavior	0.65	0.34	-0.07
Item 15: Debriefing provided a means for me to reflect on my actions during the simulation	**0.64**	**0.41**	-0.31
Item 11: The debriefing environment was physically comfortable	**0.60**	**0.49**	0.11
Item 1: Debriefing helped me to make connections in my learning	**0.60**	**0.45**	-0.53
Item 4: Debriefing helped me to find meaning in the simulation	**0.59**	**0.42**	-0.30
Item 8: Debriefing helped me to make connections between theory and real-life situations	0.57	0.31	-0.29
Item 3: Debriefing provided me with a learning opportunity	0.54	0.39	-0.35
Item 18: The facilitator taught the right amount during the debriefing session	**0.54**	**0.77**	-0.12
Item 14: The debriefing session facilitator talked the right amount during debriefing	0.38	0.70	-0.28
Item 17: The debriefing session facilitator was an expert in the content area	0.31	0.70	-0.20
Item 16: I had enough time to debrief thoroughly	**0.46**	**0.67**	-0.19
Item 6: I became more aware of myself during the debriefing session	0.35	0.64	0.00
Item 13: The facilitator allowed me enough time to verbalize my feelings before commenting	0.38	**0.58**	**-0.68**
Item 7: Debriefing helped me to clarify problems	0.41	0.30	-0.68

Extraction method: Principal Component Analysis

Rotation method: Oblimin with Kaiser Normalization

**Fig. 2:**
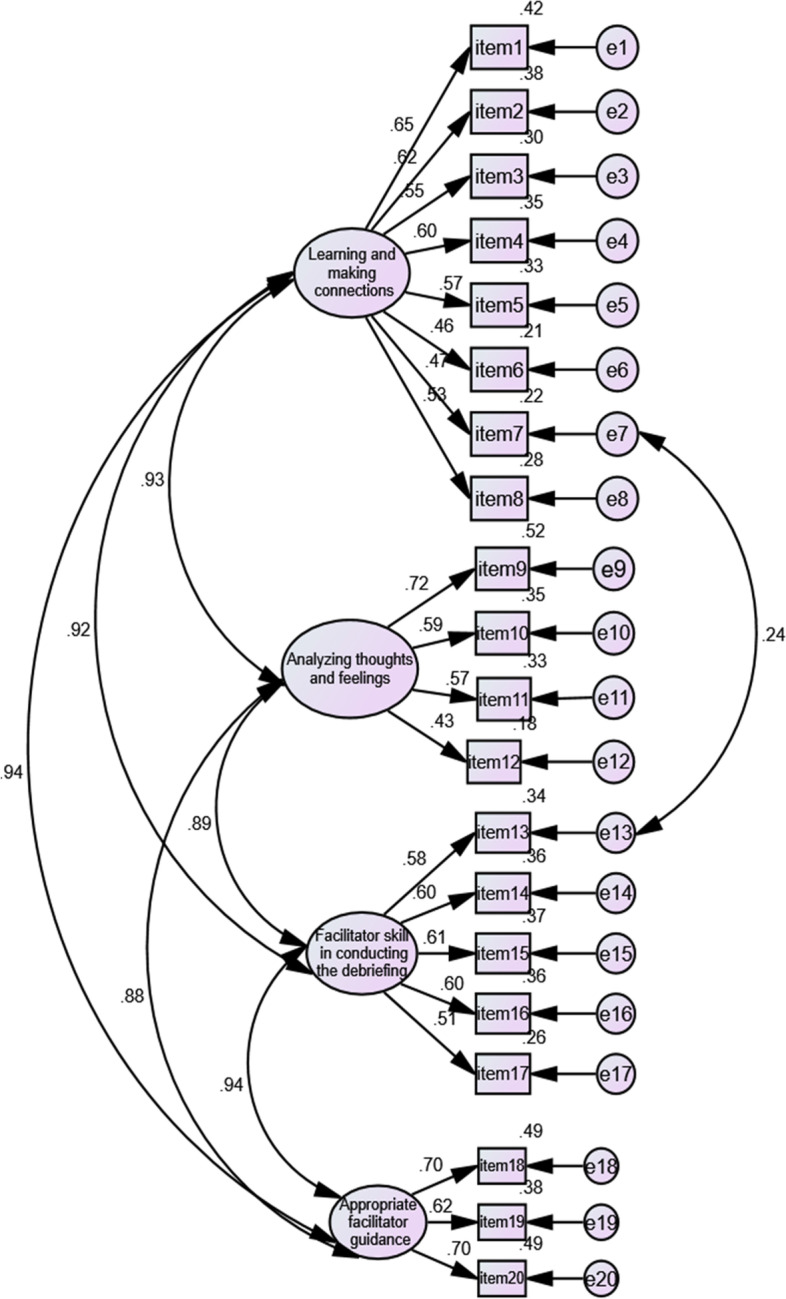
CFA of the simplified Chinese version of the DES

Confirmatory factor analysis (CFA) was implemented by using Amos Graphics (version 22). The simplified Chinese DES followed the division established by the original version. The parameter estimates of the CFA of the simplified Chinese version of the DES are shown in Fig. 2. The entire standardized factor loading was statistically significant and greater than 0.40. All the items loaded significantly onto their respective factors. The Chi-square degree of freedom ratio (*χ*^*2*^/*df*) was 1.65, the comparative fit index (CFI) was 0.91, the root mean square error of approximation (RMSEA) was 0.05, and the incremental fit index (IFI) was 0.91.

## Discussion

The aim of the current study was to translate and validate the DES in a simplified Chinese context. Psychometric tests showed that each item of the experience scale had a good degree of discrimination, so all items were retained in the simplified Chinese version. Cronbach’s alpha for the scale of six simulation cases were between 0.81–0.91, indicating excellent reliability [26]; despite the limited sample size, data from each case were reliable. The ICC was 0.86 (95% CI 0.64–0.98) indicating good reliability [23], indicating that data collected from the six simulation cases were reliable for analysis [26, 27]. The simplified Chinese version DES showed good potential in discriminating nursing students’ experiences of debriefing. This is consistent with the original version [15], the translated Norwegian version [18], and the Portuguese version [19]. The reliability of the simplified Chinese DES was confirmed by the medium to high Cronbach's alpha coefficients as well, except for the subscale "Analyzing Thoughts and Feelings". It was much like the result of the Portuguese version [19]. In the Norwegian version [18], the Cronbach’ alpha coefficient for the subscale “facilitator skill in conducting the debriefing” was 0.44 and for the total scale was 0.86. After two items were removed, an improved alpha value of 0.91 and 0.66 was noted for the total scale and the subscale, respectively.

The CVI for the scale was high, indicating that the experts agreed that the items were suitable and relevant to assess the experience of debriefing and have a close relationship with the sense of the experience. The correlation coefficient between each item and the total score proved this well.

The result of the EFA showed the translated scale could be divided into three factors, diverging from the original scale. Discrepancy is commonly seen when testing the factor structure of a scale within a different cultural context [22]. Previous researchers indicated the scale would benefit from reducing the subscales [18, 19]. In this study, EFA with Oblimin rotation found a quite unexpected grouping and item 12 “Unsettled feelings from the simulation were resolved by debriefing” was suggested for deletion. There was no apparent connection among the groupings, and they cannot be renamed, so it was decided to follow the division established by the original version as well as the Portuguese version [19]. A possible explanation for this divergence was that facilitators may have emphasized the objectives of the simulation rather than students’ emotions.

Overall, there is sufficient reliability and validity evidence to support the use of the simplified Chinese DES in Chinese nursing simulation-based education. The researchers believe that the translated DES will offer an opportunity to explore the participants’ experience of debriefing in a Chinese context. Using the simplified Chinese DES in the regular evaluation of debriefing in simulation may make a significant contribution to the development of best practices in debriefing after simulation in nursing and medical education in China.

## Limitations

In this study nursing students participated in six simulations and completed questionnaires following the debriefing phase. The experience of different simulations may result in confusion as some experiences may bring back memories from an earlier debriefing that may enhance or reduce the intensity of the experience. Researchers should offer adequate time for students to complete any questionnaires after debriefings.

Another limitation of the current study may be the size of the sample, although the sample number was acceptable according to Comrey and Lee [28]. A larger sample could have resulted in a different factor solution by offering an improved subject-to-item ratio [29]. The participants were from the same school, thus a multicenter research study is needed in the future, but maintaining homogeneity of the simulation and debriefing needs to be considered.

## Conclusions

The validation process for the simplified Chinese version of the Debriefing Experience Scale showed that the scale was effective in evaluating the experience of debriefing. The result of the EFA suggested the inclusion of fewer subscales. As validity testing is an ongoing process, a larger sample size and multicenter research will contribute to consolidation of the scale’s validity.

## Supplementary Information

**Additional file 1: Supplemental Table 1. **Cronbach’s alpha for the simplified Chinese DES for six simulation cases.

## Data Availability

The datasets used during the study are available from the corresponding author upon reasonable request.

## Abbreviations

DES: Debriefing Experience Scale
NA: Not applicable
NLN: National League for Nursing
INACSL: International Nursing Association for Clinical Simulation and Learning
GAS: Gather-Analysis-Summary
ICC: Intraclass correlation coefficient
CI: Confident intervals
EFA: Exploratory factor analysis
CFA: Confirmatory factor analysis
CVI: Content validity index
KMO: Kaiser–Meyer–Olkin
CFI: Comparative fit index
RMSEA: Root mean square error of approximation
IFI: Incremental fit index
