# Supplementary material for: Cross-cultural validation and psychometric testing of the Debriefing Experience Scale (DES): a cross-sectional study
Source: BMC Med Educ. 2022 Apr 13;22:272. doi: 10.1186/s12909-022-03332-8 (PMC9006577; doi:10.1186/s12909-022-03332-8)
Supplement: Supplementary file 1 — Additional file 1: Supplemental Table 1. Cronbach’s alpha for the simplified Chinese DES for six simulation cases. [file 12909_2022_3332_MOESM1_ESM.docx]

Supplemental Table 1

| Cronbach’s alpha for the simplified Chinese DES for six simulation cases | | | | | | | |
| --- | --- | --- | --- | --- | --- | --- | --- |
|  |  | Case 1 (n=33) | Case 2 (n=34) | Case 3 (n=34) | Case 4 (n=32) | Case 5 (n=33) | Case 6 (n=34) |
| Subscale | Learning and Making Connections | 0.81 | 0.76 | 0.72 | 0.74 | 0.79 | 0.78 |
|  | Analyzing Thoughts and Feelings | 0.59 | 0.70 | 0.71 | 0.67 | 0.60 | 0.60 |
|  | Facilitator Skill in Conducting the Debriefing | 0.65 | 0.74 | 0.80 | 0.72 | 0.72 | 0.67 |
|  | Appropriate Facilitator Guidance | 0.76 | 0.77 | 0.67 | 0.78 | 0.61 | 0.62 |
| Overall scale |  | 0.90 | 0.91 | 0.91 | 0.89 | 0.81 | 0.90 |
